# Metagenome Sequencing of a Coastal Marine Microbial Community from Monterey Bay, California

**DOI:** 10.1128/genomeA.00341-15

**Published:** 2015-04-30

**Authors:** Ryan S. Mueller, Sam Bryson, Brandon Kieft, Zhou Li, Jennifer Pett-Ridge, Francisco Chavez, Robert L. Hettich, Chongle Pan, Xavier Mayali

**Affiliations:** a Department of Microbiology, Oregon State University, Corvallis, Oregon, USA; b Computer Science and Mathematics Division, Oak Ridge National Laboratory, Oak Ridge, Tennessee, USA; c Physical and Life Science Directorate, Lawrence Livermore National Laboratory, Livermore, California, USA; d Monterey Bay Aquarium Research Institute, Moss Landing, California, USA; e Department of Microbiology, University of Tennessee, Knoxville, Tennessee, USA; f Chemical Sciences Division, Oak Ridge National Laboratory, Oak Ridge, Tennessee, USA

## Abstract

Heterotrophic microbes are critical components of aquatic food webs. Linkages between populations and the substrates they utilize are not well defined. We present the metagenome of microbial communities from the coastal Pacific Ocean exposed to various nutrient additions in order to better understand substrate utilization and partitioning in this environment.

## GENOME ANNOUNCEMENT

Marine phytoplankton and cyanobacteria are estimated to fix approximately 50 gigatons of carbon (C) annually (1). Concomitantly, heterotrophic microbes within marine food webs on average consume roughly 50% of fixed C (2). Considering the immense size of this organic matter pool and the importance of microbes in its turnover, microbial consumers can significantly affect processes such as global climate patterns and the transfer of energy through food webs (2, 3). New approaches are beginning to provide information on the roles of key populations contributing to biogeochemical cycles in marine systems (4). Here, we used metagenomics to examine the population dynamics within microbial communities in response to the addition of organic C substrates (acetate, amino acids, glucose, lipids, protein, and starch) and to constrain resource utilization preferences of lineages based on response patterns.

Surface seawater was collected during a cruise aboard the R/V *Rachel Carson* within Monterey, Bay, California, USA (36°N 53.387′, 121°W 57.257′). Carboys were kept covered at ambient temperature until initial processing (4 to 10 h), whereupon samples were prefiltered through sterile Whatman 934-AH glass fiber filters (1.5 µm nominal pore size). Experimental treatments consisted of the substrates listed above (1 µM, final concentration), with incubations lasting ~15 h at 19°C. One liter from each of three replicates (controls and substrate additions) was filtered through 0.2-µm Pall Supor membranes in order to collect the free-living fraction. Filters were frozen and stored at −80°C until DNA was extracted (MasterPure Kit; Epicentre Technologies). DNA from replicate samples was pooled to create one library (Illumina TruSeq kit; Illumina, Inc.) for each treatment (*n* = 7). Sequencing of all libraries was performed using the Illumina MiSeq platform version 3.0. After quality filtering of reads, the total generated sequence from each library was as follows: control, 10.5 Gb; acetate, 1.6 Gb; amino acids, 1.4 Gb; glucose, 2.5 Gb; lipids, 1.5 Gb; protein, 2.2 Gb; and starch, 1.5 Gb.

Assemblies were optimized using multiple programs (Velvet [5], IDBA [6], MaSuRCA [7], Newbler [8], and Ray [9]), and resulting contigs were merged into one final assembly for each metagenome using GAM-NGS (10). Dominant phyla within metagenomes were *Proteobacteria* (31% to 56%), *Bacteroidetes* (26% to 61%), *Verrucomicrobia* (3% to 5%), *Euryarchaeota* (0% to 3%), *Thaumarchaeota* (0% to 2%), *Planctomycetes* (0% to 2%), *Actinobacteria* (0% to 2%), *Marinomicrobia* (0% to 2%), and *Cyanobacteria* (0% to 2%). Specific populations significantly increased in abundance in response to different substrate additions (e.g., relative abundances of *Marinimicrobia* and *Roseobacter* populations increased in lipid and acetate treatments, respectively), while other taxa demonstrated the same general trend across all treatments (e.g., *Acidobacteria*, marine group II *Euryarchaeota*, and *Planctomycetes* populations decreased in abundance in all treatments, while *Polaribacter* populations increased).

The reported metagenomes provide detailed information on individual population dynamics in response to nutrient addition, allowing for insight into the membership of functional guilds within marine microbial food webs.

### Nucleotide sequence accession numbers. 

DNA sequences from this project were deposited under the accession number SRR1873745 within the NCBI SRA and identification number 4622002.3 within the MG-RAST server.

## References

[1] Hedges JI, Oades JM 1997 Comparative organic geochemistries of soils and marine sediments. Org Geochem 27:319–361. doi:10.1016/S0146-6380(97)00056-9.

[2] Kirchman DL (ed) 2008 Microbial ecology of the oceans, 2nd ed. John Wiley & Sons, Hoboken, NJ.

[3] Howard EC, Sun S, Biers EJ, Moran MA 2008 Abundant and diverse bacteria involved in DMSP degradation in marine surface waters. Environ Microbiol 10:2397–2410. doi:10.1111/j.1462-2920.2008.01665.x. 18510552

[4] Béjà O, Spudich EN, Spudich JL, Leclerc M, DeLong EF 2001 Proteorhodopsin phototrophy in the ocean. Nature 411:786–789. doi:10.1038/35081051. 11459054

[5] Zerbino DR, Birney E 2008 Velvet: algorithms for *de novo* short read assembly using de Bruijn graphs. Genome Res 18:821–829. doi:10.1101/gr.074492.107. 18349386 PMC2336801

[6] Peng Y, Leung HC, Yiu SM, Chin FY 2012 IDBA-UD: a *de novo* assembler for single-cell and metagenomic sequencing data with highly uneven depth. Bioinformatics 28:1420–1428. doi:10.1093/bioinformatics/bts174. 22495754

[7] Zimin AV, Marçais G, Puiu D, Roberts M, Salzberg SL, Yorke JA 2013 The MaSuRCA genome assembler. Bioinformatics 29:2669–2677. doi:10.1093/bioinformatics/btt476. 23990416 PMC3799473

[8] Margulies M, Egholm M, Altman WE, Attiya S, Bader JS, Bemben LA, Berka J, Braverman MS, Chen YJ, Chen Z, Dewell SB, Du L, Fierro JM, Gomes XV, Godwin BC, He W, Helgesen S, Ho CH, Irzyk GP 2005 Genome sequencing in microfabricated high-density picolitre reactors. Nature 437:376–380. doi:10.1038/nature03959. 16056220 PMC1464427

[9] Boisvert S, Raymond F, Godzaridis E, Laviolette F, Corbeil J 2012 Ray Meta: scalable *de novo* metagenome assembly and profiling. Genome Biol 13:R122. doi:10.1186/gb-2012-13-12-r122. 23259615 PMC4056372

[10] Vicedomini R, Vezzi F, Scalabrin S, Arvestad L, Policriti A 2013 GAM-NGS: genomic assemblies merger for next generation sequencing. BMC Bioinformatics 14(Suppl 7):S6. doi:10.1186/1471-2105-14-S7-S6. 23815503 PMC3633056

